# Prognostic Value of Baseline Systemic Immune-Inflammation Index in Advanced Intrahepatic Cholangiocarcinoma Treated with First-Line Gemcitabine–Cisplatin Plus PD-L1 Inhibitor: A Single-Center Retrospective Study

**DOI:** 10.3390/curroncol33020123

**Published:** 2026-02-19

**Authors:** Shuan Wu, Jiawei Xu, Yan Li, Decai Yu

**Affiliations:** Division of Hepatobiliary and Transplantation Surgery, Department of General Surgery, Nanjing Drum Tower Hospital, Affiliated Hospital of Medical School, Nanjing University, Nanjing 210008, China

**Keywords:** intrahepatic cholangiocarcinoma, systemic immune-inflammation index, gemcitabine, cisplatin, PD-L1 inhibitor, prognosis, objective response rate

## Abstract

Background: Gemcitabine–cisplatin (GC) combined with a programmed death-ligand 1 (PD-L1) inhibitor has become an important first-line regimen for advanced intrahepatic cholangiocarcinoma (ICC). However, overall efficacy remains modest, and inter-patient heterogeneity in outcomes is substantial, highlighting the need for simple biomarkers for pretreatment risk stratification. The systemic immune-inflammation index (SII), derived from peripheral neutrophil, lymphocyte, and platelet counts, has been associated with prognosis in various malignancies, but its clinical relevance in advanced ICC treated with first-line GC plus PD-L1 inhibitor remains unclear. Aims: To evaluate the association of baseline SII with objective response and survival outcomes in patients with advanced ICC receiving first-line GC plus PD-L1 inhibitor. Methods: We retrospectively analyzed 193 consecutive patients with advanced ICC who received first-line GC plus a PD-L1 inhibitor at our center. Baseline clinicopathologic characteristics and laboratory parameters were collected, and SII was calculated as platelet count (×10^9^/L) × neutrophil count (×10^9^/L)/lymphocyte count (×10^9^/L). Receiver operating characteristic (ROC) analysis was performed to assess the discriminative ability of baseline SII for objective response and to determine an internally derived cut-off value. Patients were categorized into low- and high-SII groups accordingly. Logistic regression was used to identify factors associated with objective response rate (ORR). Progression-free survival (PFS) and overall survival (OS) were estimated by the Kaplan–Meier method and compared using the log-rank test. Multivariable Cox proportional hazards models were constructed to evaluate the independent prognostic significance of SII for PFS and OS. Results: Among the 193 patients included, 55 achieved complete or partial response and 138 had stable or progressive disease, yielding an ORR of 28.5%. Baseline SII showed good discrimination for objective response (AUC = 0.91), and the optimal cut-off value was 495.75. Patients in the low-SII group had a significantly higher ORR than those in the high-SII group (*p* < 0.001). Kaplan–Meier analysis demonstrated that both PFS and OS were longer in the low-SII group than in the high-SII group (median OS: 13.0 vs. 8.0 months, log-rank *p* < 0.001; median PFS: 8.5 vs. 6.0 months, *p* = 0.025). In multivariable Cox models adjusting for differentiation, CA19-9, tumor multiplicity, and distant metastasis, SII grouping remained independently associated with PFS and OS, and distant metastasis was consistently associated with increased risks of progression and death. Conclusions: Baseline SII is a readily available prognostic biomarker associated with objective response and survival in patients with advanced ICC treated with first-line GC plus PD-L1 inhibitor. Given the retrospective single-center design, the absence of a non-immunotherapy comparator cohort, and internal cut-off derivation, these findings should be interpreted as hypothesis-generating and warrant external validation.

## Simple Summary

Bile duct cancer that starts inside the liver is difficult to treat, and people can respond very differently to the same medicines. Doctors need simple and inexpensive ways to identify patients who may do better or worse before treatment begins. In this study, we reviewed the medical records of 193 patients who received first-line treatment with two standard chemotherapy drugs (gemcitabine and cisplatin) together with durvalumab, a medicine that helps the immune system attack cancer. We used routine blood tests taken before treatment to create an index that reflects the balance between inflammation and immune strength. Patients with a lower index were more likely to have their tumors shrink on scans, and they lived longer without the cancer getting worse, as well as longer overall, compared with patients who had a higher index. This blood-based measure is easy to obtain in everyday practice and could help doctors counsel patients, plan follow-up, and identify those who may need closer monitoring or additional treatment strategies. Because this was a single-hospital study looking back at on existing records, the findings require confirmation in other groups of patients.

## 1. Introduction

Intrahepatic cholangiocarcinoma (ICC) is the second most frequent primary liver cancer following hepatocellular carcinoma, and it is commonly detected at an advanced stage, when curative resection is no longer possible [1,2,3,4]. For many years, the combination of gemcitabine and cisplatin (GC) has been the standard first-line systemic therapy for advanced biliary tract cancers, including ICC [5,6]. More recently, the addition of PD-L1 inhibitors to GC has improved survival in phase III trials, establishing GC plus PD-L1 blockade as a new standard-of-care regimen for many patients with advanced ICC [7,8]. Nevertheless, the overall survival benefit remains limited, and there is marked heterogeneity in treatment response and prognosis among individuals. Identifying simple and reliable biomarkers to facilitate pretreatment risk stratification and to better characterize outcome heterogeneity under chemo-immunotherapy, therefore, remains an important unmet clinical need [9,10,11].

The current assessment of efficacy and prognosis in advanced ICC treated with chemo-immunotherapy primarily relies on serial imaging according to RECIST criteria and, in selected cases, on tumor tissue or molecular biomarkers. However, tissue-based and genomic markers are often costly, require invasive sampling, and are not widely available in routine practice. In contrast, inflammation-based indices derived from routine blood tests have attracted increasing attention. Calculated using platelets, neutrophils, and lymphocytes (platelet count × neutrophil count/lymphocyte count), the systemic immune-inflammation index (SII) serves as a composite indicator of inflammatory activity and immune function [12]. Accumulating evidence suggests that elevated SII is associated with poor prognosis in various solid tumors, including hepatocellular carcinoma and other gastrointestinal cancers [12,13,14,15,16,17].

Several studies have evaluated the prognostic role of SII in patients with ICC or biliary tract cancers treated with surgery, liver transplantation, or locoregional therapies [18,19]. Data regarding the predictive and prognostic value of SII in advanced ICC, particularly in the context of first-line chemo-immunotherapy combining GC with PD-L1 inhibition, are scarce [18,19,20,21,22]. It remains unclear whether baseline SII can identify patients who are more likely to respond to such regimens and to achieve prolonged survival. In this single-center retrospective study, we aimed to evaluate the association between baseline SII and treatment response, progression-free survival (PFS), and overall survival (OS) in patients with advanced ICC receiving first-line GC plus a PD-L1 inhibitor, and to explore the potential role of SII as a practical biomarker for pretreatment risk stratification and individualized treatment planning.

## 2. Materials and Methods

### 2.1. Study Design and Patients

This was a single-center retrospective cohort study. We screened consecutive patients with advanced ICC who received first-line GC plus a PD-L1 inhibitor as systemic therapy at our institution between January 2023 and October 2025. Inclusion criteria were: (1) pathologically or radiologically confirmed ICC; (2) unresectable or recurrent disease not amenable to curative surgery or local ablative therapies; (3) first-line systemic treatment with GC combined with a PD-L1 inhibitor; (4) at least one measurable lesion according to RECIST 1.1 [23]; and (5) available baseline clinical and laboratory data as well as follow-up information. Exclusion criteria included: (1) prior systemic anti-tumor therapy (chemotherapy, targeted therapy, or immunotherapy); (2) concurrent malignancies; (3) active infection, autoimmune disease, or hematologic disorders that could significantly affect blood counts; and (4) data with severely missing variables or outcomes.

The study protocol was conducted in accordance with the principles of the Declaration of Helsinki and was approved by the institutional ethics committee of our hospital (approval No. 2025-067). Given the retrospective design and use of anonymized data, the requirement for written informed consent was waived.

### 2.2. Treatment Regimen

All patients received first-line GC plus a PD-L1 inhibitor. GC was administered according to standard schedules, with gemcitabine given intravenously on days 1 and 8 and cisplatin on day 1 of each 21-day cycle. The PD-L1 inhibitor was administered intravenously at the recommended dose on day 1 of each cycle. Treatment was continued until radiologically confirmed disease progression, unacceptable toxicity, withdrawal of consent, or physician decision. Dose modifications and treatment delays were permitted based on toxicity and clinical judgment, in line with routine clinical practice.

In routine clinical practice, maintenance durvalumab following completion of gemcitabine–cisplatin was administered at the discretion of the treating physician. Patients without disease progression after induction therapy generally continued durvalumab monotherapy every 4 weeks, in accordance with the maintenance strategy used in the TOPAZ-1 trial. However, some patients discontinued immunotherapy due to toxicity, patient preference, or other clinical considerations.

### 2.3. Data Collection and Variable Definitions

Electronic medical records were reviewed to collect baseline characteristics, such as patient age, sex, and ECOG performance status, along with other clinical and tumor-related variables: tumor differentiation (well/moderately vs. poorly differentiated), tumor multiplicity (single vs. multiple lesions), maximum tumor diameter, presence of vascular invasion, lymph node involvement, and distant metastasis. Laboratory parameters at baseline included complete blood counts (white blood cells, neutrophils, lymphocytes, and platelets), liver function tests, lactate dehydrogenase (LDH), bilirubin, albumin, and tumor markers (e.g., CA19-9). CA19-9 was dichotomized at 400 U/mL and maximum tumor diameter at 7 cm to reflect markedly elevated tumor burden while preserving adequate subgroup sizes for analysis.

SII was calculated from peripheral blood counts using the formula platelet count (×10^9^/L) × neutrophil count (×10^9^/L)/lymphocyte count (×10^9^/L). Measurements obtained within 7 days before the first cycle of GC plus PD-L1 inhibitor were used to represent baseline SII. Additional indices were determined based on standard definitions.

### 2.4. Efficacy Assessment and Follow-Up

Tumor response was assessed using contrast-enhanced computed tomography (CT) or magnetic resonance imaging (MRI) and evaluated in accordance with RECIST version 1.1. All imaging studies were independently interpreted by two experienced radiologists; any discrepancies were adjudicated through consensus with the involvement of a third radiologist. Responses were classified as complete response (CR), partial response (PR), stable disease (SD), or progressive disease (PD), and the objective response rate (ORR) was calculated as the proportion of patients achieving CR or PR.

Follow-up commenced on the date of initiation of gemcitabine–cisplatin plus PD-L1 inhibitor therapy. Progression-free survival (PFS) was defined as the interval from treatment start to radiologically confirmed disease progression or death from any cause, whichever occurred first. Overall survival (OS) was defined as the time from treatment initiation to death from any cause; patients who were alive at the last follow-up were censored. Survival status was ascertained through outpatient visits and telephone follow-up, with the censoring date set at 31 October 2025.

### 2.5. Statistical Analysis

Statistical analyses were conducted using SPSS version 27.0 (IBM Corp., Armonk, NY, USA) or comparable statistical software. Continuous variables are reported as mean ± standard deviation or median (with interquartile range), as appropriate according to data distribution, and were compared using Student’s *t*-test or the Mann–Whitney U test. Categorical variables are presented as counts (percentages) and were compared using the chi-square test or Fisher’s exact test, as applicable.

Receiver operating characteristic (ROC) curve analysis was performed to assess the discriminative ability of baseline SII for objective response. The optimal SII cut-off was selected by maximizing the Youden index, and patients were subsequently stratified into low-SII and high-SII groups. Univariable and multivariable logistic regression analyses were used to explore factors associated with objective response; variables with *p* < 0.10 in univariable analyses were entered into the multivariable model.

Progression-free survival (PFS) and overall survival (OS) were estimated using the Kaplan–Meier method and compared between groups with the log-rank test. Cox proportional hazards models were constructed to identify independent prognostic factors for PFS and OS. Prespecified covariates included SII group, tumor differentiation, CA19-9 (≥400 vs. <400 U/mL), tumor multiplicity, and distant metastasis. Results are reported as hazard ratios (HRs) with 95% confidence intervals (CIs). All statistical tests were two-sided, and *p* < 0.05 was considered statistically significant. No formal sample size calculation was performed a priori; the final sample size was determined by the number of eligible patients available during the study period.

## 3. Results

### 3.1. Patient Characteristics

**Figure 1:**
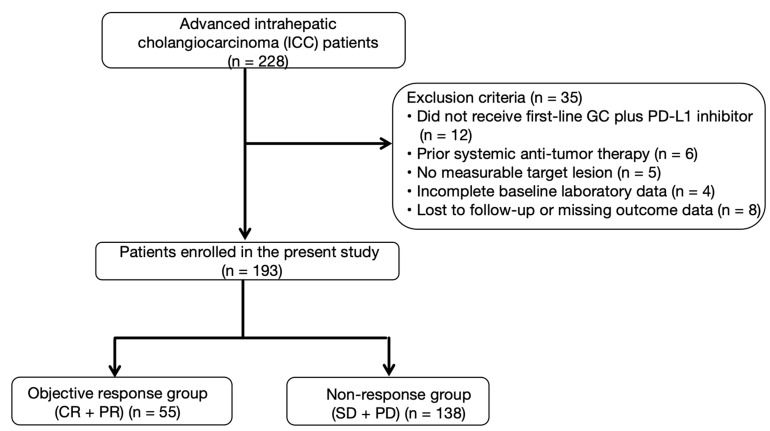
Flowchart of patient selection and overall treatment response. Flowchart summarizing patient selection and treatment response in the study cohort. A total of 228 patients with advanced intrahepatic cholangiocarcinoma (ICC) were screened for eligibility. Patients who did not receive first-line gemcitabine–cisplatin (GC) plus a PD-L1 inhibitor, had prior systemic anti-tumor therapy, lacked measurable target lesions, or had incomplete baseline or outcome data were excluded. Finally, 193 patients were included in the analysis. Among them, 55 achieved complete or partial response (CR/PR) and 138 had stable or progressive disease (SD/PD), yielding an overall objective response rate (ORR) of 28.5%. Abbreviations: ICC, intrahepatic cholangiocarcinoma; GC, gemcitabine–cisplatin; PD-L1, programmed death-ligand 1; CR, complete response; PR, partial response; SD, stable disease; PD, progressive disease.

A total of 228 patients with advanced ICC were screened (Figure 1). Thirty-five patients were excluded because they did not receive first-line GC plus a PD-L1 inhibitor, had received prior systemic therapy, lacked measurable lesions, or had incomplete baseline or outcome data. Ultimately, 193 patients were included in the analysis. The data cut-off date was 31 October 2025. Among them, 55 patients achieved CR or PR, and 138 had SD or PD, resulting in an ORR of 28.5%.

The mean age at treatment initiation was 62.1 ± 8.7 years, with a slight predominance of males. Approximately half of the tumors were poorly differentiated, and most patients had a single dominant lesion at baseline. Around half of the cohort had intrahepatic or distant metastases. The median baseline CA19-9 level was 124.0 U/mL, and 36.5% of patients had CA19-9 ≥ 400 U/mL. The median SII was 625 (IQR 470.1–969.0), with a wide distribution across the cohort. Baseline clinical and laboratory characteristics are summarized in Table 1.

### 3.2. Baseline Hematologic Indices and SII According to Treatment Response

Of the 193 patients, 55 were classified as responders (CR/PR) and 138 as non-responders (SD/PD). Comparisons of baseline blood counts and clinical variables between these two groups are shown in Table 2. Responders had significantly lower baseline SII values than non-responders, whereas several other hematologic parameters and tumor-related factors did not differ substantially. These findings indicate that lower baseline systemic inflammatory burden and relatively preserved immune status are associated with a higher probability of achieving radiologic response to GC plus PD-L1 inhibitor therapy.

### 3.3. Association Between Baseline SII and Objective Response

**Figure 2:**
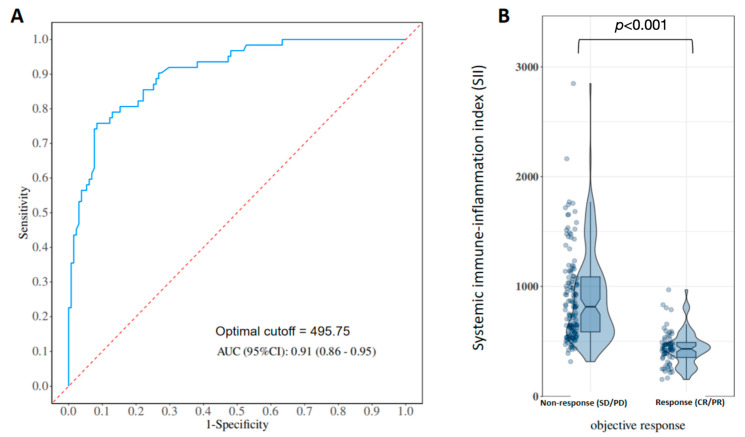
Predictive performance of baseline systemic immune-inflammation index (SII) for objective response to gemcitabine–cisplatin plus PD-L1 inhibitor. (**A**) Receiver operating characteristic (ROC) curve of baseline SII for discriminating responders (CR/PR) from non-responders (SD/PD) to first-line gemcitabine–cisplatin (GC) plus PD-L1 inhibitor. The area under the ROC curve (AUC) is 0.91, indicating high predictive accuracy. The optimal cut-off value determined by the Youden index is 495.75. (**B**) Violin plots showing the distribution of baseline SII according to response status. The overall SII distribution is shifted toward lower values in responders (CR/PR) compared with non-responders (SD/PD), with clear separation between the two groups. Abbreviations: SII, systemic immune-inflammation index; ROC, receiver operating characteristic; AUC, area under the curve; GC, gemcitabine–cisplatin; PD-L1, programmed death-ligand 1; CR, complete response; PR, partial response; SD, stable disease; PD, progressive disease.

ROC curve analysis demonstrated that baseline SII had good discriminative ability for identifying responders (Figure 2), with an AUC of 0.91. The optimal cut-off value determined by the Youden index was 495.75. Using this cut-off, patients in the low-SII group had a significantly higher ORR than those in the high-SII group (*p* < 0.001). In multivariable logistic regression analyses adjusting for sex, CA19-9 level, maximum tumor diameter, tumor multiplicity, distant metastasis, and tumor differentiation, SII grouping remained independently associated with ORR, indicating that lower baseline SII was associated with a higher likelihood of response to GC plus PD-L1 inhibitor (Supplementary Figure S1).

### 3.4. Progression-Free and Overall Survival According to SII Groups

**Figure 3:**
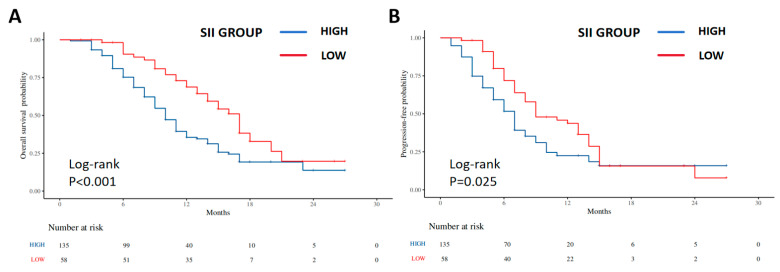
Progression-free and overall survival according to baseline SII groups. Kaplan–Meier survival curves for patients with advanced intrahepatic cholangiocarcinoma treated with first-line gemcitabine–cisplatin plus PD-L1 inhibitor, stratified by baseline systemic immune-inflammation index (SII) using the optimal cut-off of 495.75. (**A**) Overall survival (OS) in the low-SII group (SII < 495.75, *n* = 58) versus the high-SII group (SII ≥ 495.75, *n* = 135). The survival curves separate early and remain apart throughout follow-up (log-rank *p* < 0.001). (**B**) Progression-free survival (PFS) in the low-SII and high-SII groups, showing significantly longer PFS in the low-SII group (log-rank *p* = 0.025). Abbreviations: SII, systemic immune-inflammation index; OS, overall survival; PFS, progression-free survival.

When patients were stratified by the optimal SII cut-off, the low-SII group had significantly longer OS and PFS than the high-SII group (Figure 3). For OS, the median survival was 13.0 months in the low-SII group versus 8.0 months in the high-SII group, and the hazard of death was markedly reduced in the low-SII group (log-rank *p* < 0.001; HR = 0.51, 95% CI 0.33–0.77). Median PFS was 8.5 months among patients in the low-SII group, compared with 6.0 months in the high-SII group, indicating a lower hazard of progression or death for the low-SII cohort (log-rank *p* = 0.025; HR = 0.68, 95% CI 0.47–0.98).

### 3.5. Multivariable Cox Regression for PFS and OS

Variables including sex, tumor differentiation, SII group, CA19-9 (≥400 vs. <400 U/mL), maximum tumor diameter (≥7 vs. <7 cm), tumor multiplicity, and presence of distant metastasis were entered into Cox proportional hazards models for OS and PFS (Table 3). In the OS model, the SII group and distant metastasis emerged as independent prognostic factors. Compared with the high-SII group, patients in the low-SII group had a substantially reduced risk of death (HR = 0.49, 95% CI 0.32–0.75, *p* < 0.001), whereas the presence of distant metastasis was associated with an increased mortality risk (HR = 1.78, 95% CI 1.21–2.61, *p* = 0.003). In the PFS model, low SII was similarly associated with a lower risk of progression or death (HR = 0.65, 95% CI 0.45–0.95, *p* = 0.025), and distant metastasis remained an adverse prognostic factor (HR = 1.62, 95% CI 1.14–2.32, *p* = 0.008). In addition, patients with solitary tumors had a higher risk of progression compared with those with multiple lesions (HR = 1.49, 95% CI 1.02–2.18, *p* = 0.039). By contrast, sex, tumor differentiation, baseline CA19-9 ≥400 U/mL, and maximum tumor diameter ≥ 7 cm had hazard ratios close to 1 and were not retained as independent prognostic factors in the multivariable models (Table 3).

## 4. Discussion

In this single-center retrospective study of 193 patients with advanced ICC treated with first-line GC plus a PD-L1 inhibitor, we comprehensively evaluated the association and prognostic significance of baseline SII. Our main findings can be summarized as follows. First, baseline SII was significantly lower in responders than in non-responders, suggesting that patients with a lower systemic inflammatory burden and better preserved immune status are more frequently observed to achieve radiologic response under chemo-immunotherapy. Second, ROC analysis showed that SII had high accuracy for predicting ORR, with an AUC of 0.91; using an optimal cut-off value of 495.75, the low-SII group had a markedly higher ORR than the high-SII group. Third, Kaplan–Meier and Cox analyses demonstrated that patients with low baseline SII had substantially longer PFS and OS than those with high SII, and that SII grouping remained an independent prognostic factor of both endpoints after adjustment for conventional clinicopathologic variables. In contrast, several traditional tumor burden-related factors, such as CA19-9 elevation and tumor multiplicity and differentiation, did not retain independent prognostic significance in our cohort.

The biological rationale underlying the association between elevated SII and poor outcomes in cancer patients is plausible. SII integrates neutrophil, lymphocyte, and platelet counts into a single composite index reflecting the balance between systemic inflammation and host immune competence. Neutrophils and platelets can promote tumor progression through the release of cytokines, chemokines, and pro-angiogenic factors, thereby facilitating tumor growth, invasion, and immune evasion. In contrast, lymphocytes—particularly cytotoxic T cells and natural killer cells—play a central role in antitumor immune surveillance. A high SII, therefore, represents a systemic milieu characterized by inflammation and relative lymphopenia, which may be associated with impaired antitumor immunity and unfavorable treatment outcomes. Such an immune-inflammatory state may influence overall responsiveness to anticancer therapies, including chemotherapy and immune checkpoint inhibition, although the present study cannot distinguish treatment-specific effects [24,25,26].

Our findings are in line with previous studies reporting that elevated SII is associated with worse survival in various solid tumors, including hepatocellular carcinoma, gastric cancer, and pancreatic cancer, treated with surgery, chemotherapy, or locoregional therapies [27,28,29]. However, data on SII in ICC, especially in patients receiving first-line chemo-immunotherapy, have been limited. By demonstrating that baseline SII is associated with objective response as well as with PFS and OS in advanced ICC patients receiving GC plus PD-L1 inhibitor, our study extends prior observations into the real-world immunotherapy era, while supporting a primarily prognostic role of SII within a uniformly treated cohort [30,31]. The observation that SII outperformed several conventional tumor burden-related markers in multivariable models further underscores the importance of host-related systemic inflammation, in addition to tumor factors alone, in shaping treatment outcomes for advanced ICC.

Beyond ICC, several studies have explored whether SII is linked to outcomes under different systemic treatment modalities, including cytotoxic chemotherapy and immune checkpoint inhibition. In advanced non-small-cell lung cancer treated with platinum-doublet chemotherapy, a composite score integrating SII and the prognostic nutritional index (PNI) after four cycles of treatment was strongly associated with PFS and OS, with higher SII-PNI scores identifying patients with substantially worse survival, suggesting that an adverse systemic inflammatory and nutritional profile can diminish the benefit of cytotoxic chemotherapy [32]. In locally advanced gastric cancer, pre-treatment SII combined with PNI also predicted pathological response and progression-free survival in patients receiving neoadjuvant XELOX chemotherapy plus the anti-PD-1 antibody sintilimab, indicating that elevated SII is associated with poor efficacy of combined chemo-immunotherapy [33]. Furthermore, high baseline SII has been shown to correlate with inferior survival in patients with advanced gastric cancer receiving dual PD-1 and HER2 blockade, and in large real-world cohorts of pretreated urothelial carcinoma treated with atezolizumab, where SII-based immune-inflammatory scores retained independent prognostic value for OS and PFS [34]. In HER2-positive breast cancer, high SII was associated with shorter disease-free survival among patients treated with trastuzumab, whereas no clear prognostic effect was observed in those who did not receive trastuzumab, raising the possibility that systemic immune-inflammation may also modulate sensitivity to targeted antibody therapy [35]. Taken together, these data support the concept that SII mainly captures the host systemic inflammatory and immune milieu, which can influence the effectiveness of both cytotoxic chemotherapy and immunotherapy-based regimens, rather than being specific to a single therapeutic component.

We also identified distant metastasis as a consistent adverse prognostic factor for both PFS and OS, in line with the aggressive biology and limited therapeutic options associated with disseminated ICC. Together, SII and metastatic status may serve as complementary variables for pretreatment risk stratification, potentially enabling the identification of patient subgroups with distinct prognostic profiles under standard GC plus PD-L1 inhibitor therapy. Such stratification may assist clinicians in tailoring follow-up intensity, counseling patients regarding expected outcomes, and considering clinical trial enrollment for higher-risk individuals.

Several limitations of this study should be acknowledged. First, the retrospective and single-center design introduces inherent selection and information bias, and although nearly 200 patients were included, the sample size remains modest. These factors limit the generalizability of our findings, which should therefore be regarded as hypothesis-generating and warrant validation in larger, prospective, multicenter cohorts. Second, although all patients received GC plus a PD-L1 inhibitor as first-line therapy, heterogeneity in treatment duration, dose modifications, and real-world management could not be fully controlled. Third, SII was assessed only at baseline; dynamic changes in SII during treatment were not evaluated, and their potential prognostic relevance remains to be explored. Fourth, we did not directly compare SII with other inflammation-based indices, such as the neutrophil-to-lymphocyte ratio or platelet-to-lymphocyte ratio, precluding conclusions regarding the relative superiority of SII. Finally, the absence of a control cohort treated without PD-L1 inhibition precludes a differentiation between a predictive biomarker of immunotherapy benefit and a purely prognostic marker, and this distinction should be addressed in future comparative studies.

Despite these limitations, our study has important clinical implications. SII is derived from routine complete blood counts that are inexpensive, standardized, and widely available, making it particularly attractive for real-world application. The incorporation of baseline SII into pretreatment evaluation may provide a simple means of prognostic stratification in patients with advanced ICC undergoing first-line GC plus PD-L1 inhibitor therapy. Future studies integrating SII with tumor-related, molecular, and treatment-specific factors may further refine individualized prognostic assessment and therapeutic decision-making in this challenging disease [36].

## 5. Conclusions

The baseline systemic immune-inflammation index is a simple, inexpensive, and informative prognostic of treatment response and survival in patients with advanced intrahepatic cholangiocarcinoma treated with first-line gemcitabine–cisplatin plus a PD-L1 inhibitor. Patients with low SII are more likely to achieve objective response and have significantly longer progression-free and overall survival than those with high SII. In multivariable analyses, SII grouping and distant metastasis were independent prognostic factors for both PFS and OS, whereas several conventional tumor burden-related variables were not. If validated in larger multicenter prospective studies and external cohorts, SII could be incorporated into routine pretreatment risk stratification and serve as a practical biomarker to guide individualized chemo-immunotherapy strategies in advanced ICC.

## Figures and Tables

**Table 1:** Baseline clinicopathologic and laratory characteristics of patients with advanced intrahepatic cholangiocarcinoma treated with first-line gemcitabine–cisplatin plus PD-L1 inhibitor (*n* = 193).

Variable	Total (*n* = 193)
Demographic characteristics	
Sex, *n* (%)	
Male	102 (52.8)
Female	91 (47.2)
Age, years, mean ± SD	62.1 ± 8.7
Tumor characteristics	
Tumor differentiation, *n* (%)	
Medium-to-high differentiation	91 (47.2)
Poor differentiation	102 (52.8)
Longest tumor diameter ≥ 7 cm, *n* (%)	
No	129 (66.8)
Yes	64 (33.2)
Number of intrahepatic lesions, *n* (%)	
Single	146 (75.6)
Multiple	47 (24.4)
Limited within one hepatic lobe, *n* (%)	
No	127 (65.8)
Yes	66 (34.2)
Intrahepatic metastasis, *n* (%)	
No	84 (43.5)
Yes	109 (56.5)
Vascular invasion, *n* (%)	
No	147 (76.2)
Yes	46 (23.8)
Capsular invasion, *n* (%)	
No	178 (92.2)
Yes	15 (7.8)
Invasion of adjacent organs, *n* (%)	
No	115 (59.6)
Yes	78 (40.4)
Porta hepatis/retroperitoneal lymph node enlargement, *n* (%)	
No	3 (1.6)
Yes	190 (98.4)
Disease status, *n* (%)	
Locally advanced	93 (48.2)
Distant metastasis	100 (51.8)
Liver cirrhosis, *n* (%)	
No	172 (89.1)
Yes	21 (10.9)
Intrahepatic bile duct stones, *n* (%)	
No	182 (94.3)
Yes	11 (5.7)
HBV infection history, *n* (%)	
No	163 (84.5)
Yes	30 (15.5)
Tumor markers and inflammatory indices	
CA19-9, U/mL, median (IQR)	124.0 (41.3–990.3)
CA19-9 ≥ 400 U/mL, *n* (%)	70 (36.3)
AFP, ng/mL, median (IQR)	2.3 (1.3–5.1)
CEA, ng/mL, median (IQR)	2.52 (1.45–7.71)
SII, median (IQR)	625.3 (470.1–969.0)
Hematologic parameters	
White blood cell count, ×10^9^/L, median (IQR)	6.70 (5.80–7.80)
Neutrophil fraction, median (IQR)	0.67 (0.60–0.76)
Lymphocyte fraction, median (IQR)	0.21 (0.16–0.24)
Platelet count, ×10^9^/L, median (IQR)	187.0 (163.0–246.0)
Hemoglobin, g/L, median (IQR)	121.0 (113.0–135.0)
Liver function and biochemical parameters	
ALT, U/L, median (IQR)	25.5 (16.7–54.4)
AST, U/L, median (IQR)	29.8 (22.7–48.5)
LDH, U/L, median (IQR)	209.0 (183.0–270.0)
Total bilirubin, μmol/L, median (IQR)	14.4 (8.6–21.2)
Direct bilirubin, μmol/L, median (IQR)	3.4 (2.2–8.1)
Total protein, g/L, median (IQR)	68.9 (64.2–72.2)
Albumin, g/L, median (IQR)	39.5 (35.7–41.0)
C-reactive protein, mg/L, median (IQR)	9.8 (3.9–31.5)

Data are presented as mean ± standard deviation, median (interquartile range, IQR), or *n* (%) as appropriate. Adjacent organ invasion includes invasion of the diaphragm, right kidney, and gallbladder. Abbreviations: SD, standard deviation; HBV, hepatitis B virus; CA19-9, carbohydrate antigen 19-9; AFP, alpha-fetoprotein; CEA, carcinoembryonic antigen; SII, systemic immune-inflammation index; ALT, alanine aminotransferase; AST, aspartate aminotransferase; LDH, lactate dehydrogenase; IQR, interquartile range.

**Table 2:** Comparison of baseline clinicopathologic and laboratory characteristics between responders (CR/PR) and non-responders (SD/PD) to first-line gemcitabine–cisplatin plus PD-L1 inhibitor (*n* = 193).

Variable	Total (*n* = 193)	CR/PR (*n* = 55)	SD/PD (*n* = 138)	Statistic	*p* Value
Age and laboratory parameters					
Age, years, mean ± SD	62.13 ± 8.72	63.18 ± 8.63	61.72 ± 8.75	*t* = 1.05	0.293
White blood cell count, ×10^9^/L, median (Q1, Q3)	6.70 (5.80, 7.80)	7.00 (5.90, 7.50)	6.40 (5.70, 8.07)	Z = −0.70	0.485
Neutrophil count, ×10^9^/L, median (Q1, Q3)	4.50 (3.70, 5.60)	4.20 (3.70, 5.00)	4.50 (3.70, 6.15)	Z = −1.73	0.084
Platelet count, ×10^9^/L, median (Q1, Q3)	187.00 (163.00, 246.00)	181.00 (163.00, 214.00)	189.00 (163.00, 259.00)	Z = −1.50	0.133
SII, median (Q1, Q3)	625.27 (470.12, 969.00)	492.00 (385.20, 646.67)	726.00 (520.42, 1074.27)	Z = −5.06	<0.001
Lymphocyte count, ×10^9^/L, median (Q1, Q3)	1.40 (1.00, 1.60)	1.50 (1.20, 1.70)	1.30 (1.00, 1.50)	Z = −3.78	<0.001
Monocyte count, ×10^9^/L, median (Q1, Q3)	0.40 (0.30, 0.60)	0.40 (0.30, 0.60)	0.40 (0.30, 0.50)	Z = −0.58	0.562
Red blood cell count, ×10^12^/L, median (Q1, Q3)	3.96 (3.76, 4.39)	3.95 (3.77, 4.09)	4.06 (3.76, 4.49)	Z = −1.14	0.256
ALT, U/L, median (Q1, Q3)	25.50 (16.70, 54.40)	29.50 (20.30, 50.90)	25.50 (16.30, 60.60)	Z = −0.18	0.856
AST, U/L, median (Q1, Q3)	29.80 (23.50, 48.50)	29.00 (23.25, 52.50)	29.40 (22.47, 48.33)	Z = −0.74	0.46
LDH, U/L, median (Q1, Q3)	209.00 (183.00, 270.00)	232.00 (184.00, 280.00)	206.00 (182.25, 270.00)	Z = −0.55	0.58
Total bilirubin, μmol/L, median (Q1, Q3)	14.40 (8.60, 21.20)	13.60 (9.10, 20.20)	12.85 (8.50, 21.30)	Z = −1.05	0.293
Direct bilirubin, μmol/L, median (Q1, Q3)	3.40 (2.20, 8.10)	3.90 (2.25, 8.10)	2.95 (2.20, 8.25)	Z = −0.95	0.341
Total protein, g/L, median (Q1, Q3)	68.90 (64.20, 72.20)	66.80 (64.20, 72.20)	68.90 (64.40, 72.20)	Z = −0.27	0.785
Albumin, g/L, median (Q1, Q3)	39.50 (35.70, 41.00)	38.00 (35.70, 41.30)	39.50 (35.70, 41.00)	Z = −1.04	0.298
C-reactive protein, mg/L, median (Q1, Q3)	9.80 (3.90, 31.50)	11.60 (4.05, 33.40)	9.80 (3.80, 31.50)	Z = −0.75	0.452
AFP, ng/mL, median (Q1, Q3)	2.30 (1.30, 5.10)	2.65 (1.83, 5.18)	2.10 (1.30, 5.00)	Z = −1.45	0.147
CA19-9, U/mL, median (Q1, Q3)	124.00 (41.30, 990.25)	113.00 (28.90, 582.00)	137.00 (41.60, 996.75)	Z = −0.97	0.332
Clinicopathologic characteristics					
Sex, *n* (%)				χ^2^ = 8.14	0.004
Male	102 (52.85)	38 (69.09)	64 (46.38)		
Female	91 (47.15)	17 (30.91)	74 (53.62)		
AJCC stage, *n* (%)				χ^2^ = 0.00	1
IIIB	8 (7.77)	2 (8.33)	6 (7.59)		
IV	95 (92.23)	22 (91.67)	73 (92.41)		
Tumor differentiation, *n* (%)				χ^2^ = 5.10	0.024
Medium-to-high differentiation	91 (47.15)	33 (60.00)	58 (42.03)		
Poor differentiation	102 (52.85)	22 (40.00)	80 (57.97)		
HBV infection history, *n* (%)				χ^2^ = 2.31	0.129
No	163 (84.46)	43 (78.18)	120 (86.96)		
Yes	30 (15.54)	12 (21.82)	18 (13.04)		
Maximum tumor diameter ≥ 7 cm, *n* (%)				χ^2^ = 7.79	0.005
No	129 (66.84)	45 (81.82)	84 (60.87)		
Yes	64 (33.16)	10 (18.18)	54 (39.13)		
Intrahepatic metastasis, *n* (%)				χ^2^ = 0.89	0.345
No	84 (43.52)	21 (38.18)	63 (45.65)		
Yes	109 (56.48)	34 (61.82)	75 (54.35)		
Vascular invasion, *n* (%)				χ^2^ = 0.50	0.479
No	147 (76.17)	40 (72.73)	107 (77.54)		
Yes	46 (23.83)	15 (27.27)	31 (22.46)		
Capsular invasion, *n* (%)				χ^2^ = 0.53	0.465
No	178 (92.23)	49 (89.09)	129 (93.48)		
Yes	15 (7.77)	6 (10.91)	9 (6.52)		
Diaphragm/right kidney/gallbladder invasion, *n* (%)				χ^2^ = 0.81	0.368
No	115 (59.59)	30 (54.55)	85 (61.59)		
Yes	78 (40.41)	25 (45.45)	53 (38.41)		
Distant metastasis, *n* (%)				χ^2^ = 2.06	0.151
No	93 (48.19)	31 (56.36)	62 (44.93)		
Yes	100 (51.81)	24 (43.64)	76 (55.07)		

Data are presented as mean ± standard deviation, median (interquartile range, IQR), or *n* (%) as appropriate. *t*-values are from independent-samples *t*-tests. Z values are from Mann–Whitney U tests. χ^2^ values are from Pearson’s χ^2^ tests; Fisher’s exact test was used when appropriate. Abbreviations: SD, standard deviation; CR, complete response; PR, partial response; SD, stable disease; PD, progressive disease; AJCC, American Joint Committee on Cancer; HBV, hepatitis B virus; CA19-9, carbohydrate antigen 19-9; AFP, alpha-fetoprotein; SII, systemic immune-inflammation index; ALT, alanine aminotransferase; AST, aspartate aminotransferase; LDH, lactate dehydrogenase; IQR, interquartile range.

**Table 3:** Univariate and multivariate Cox regression analyses of baseline factors associated with overall survival (OS) and progression-free survival (PFS).

Variable	Category	OS Univariate HR (95% CI)	*p*	OS Multivariate HR (95% CI)	*p*	PFS Univariate HR (95% CI)	*p*	PFS Multivariate HR (95% CI)	*p*
Sex	Female	1.00 (Reference)	–	1.00 (Reference)	–	1.00 (Reference)	–	1.00 (Reference)	–
	Male	0.75 (0.52–1.07)	0.115	0.70 (0.47–1.04)	0.078	0.86 (0.61–1.20)	0.363	0.73 (0.51–1.06)	0.1
Tumor differentiation	Medium-to-high	1.00 (Reference)	–	1.00 (Reference)	–	1.00 (Reference)	–	1.00 (Reference)	–
	Poor	1.41 (0.98–2.03)	0.067	1.31 (0.91–1.91)	0.151	1.16 (0.83–1.62)	0.378	1.12 (0.79–1.59)	0.512
SII group	High	1.00 (Reference)	–	1.00 (Reference)	–	1.00 (Reference)	–	1.00 (Reference)	–
	Low	0.51 (0.33–0.77)	0.002	0.49 (0.32–0.75)	<0.001	0.68 (0.47–0.97)	0.036	0.65 (0.45–0.95)	0.025
CA19-9 ≥ 400 U/mL	No (<400)	1.00 (Reference)	–	1.00 (Reference)	–	1.00 (Reference)	–	1.00 (Reference)	–
	Yes (≥400)	0.81 (0.55–1.20)	0.298	0.83 (0.56–1.24)	0.372	1.16 (0.82–1.64)	0.41	1.06 (0.73–1.52)	0.769
Maximum tumor diameter ≥ 7 cm	No	1.00 (Reference)	–	1.00 (Reference)	–	1.00 (Reference)	–	1.00 (Reference)	–
	Yes	1.28 (0.87–1.89)	0.201	1.07 (0.70–1.63)	0.761	1.20 (0.84–1.73)	0.313	0.97 (0.64–1.46)	0.872
Tumor number	Multiple	1.00 (Reference)	–	1.00 (Reference)	–	1.00 (Reference)	–	1.00 (Reference)	–
	Solitary	1.18 (0.82–1.71)	0.373	1.18 (0.78–1.78)	0.441	1.55 (1.09–2.20)	0.015	1.49 (1.02–2.18)	0.039
Distant metastasis	No	1.00 (Reference)	–	1.00 (Reference)	–	1.00 (Reference)	–	1.00 (Reference)	–
	Yes	1.60 (1.10–2.32)	0.014	1.78 (1.21–2.61)	0.003	1.60 (1.13–2.26)	0.007	1.62 (1.14–2.32)	0.008

Hazard ratios (HRs) and 95% confidence intervals (CIs) were estimated using Cox proportional hazards models. Variables with *p* < 0.10 in univariate analyses or considered clinically relevant were entered into multivariate models. An HR < 1 indicates a reduced risk of death (for OS) or progression/death (for PFS), whereas an HR > 1 indicates an increased risk compared with the reference category. Abbreviations: OS, overall survival; PFS, progression-free survival; HR, hazard ratio; CI, confidence interval; SII, systemic immune-inflammation index; CA19-9, carbohydrate antigen 19-9.

## Data Availability

The data presented in this study are available on reasonable request from the corresponding authors. The data are not publicly available due to patient privacy and ethical restrictions.

## Supplementary Materials

The following supporting information can be downloaded at: https://www.mdpi.com/article/10.3390/curroncol33020123/s1, Figure S1: Forest plot of multivariable logistic regression for factors associated with objective response (CR/PR vs. SD/PD).
