# Supplementary material for: Prognostic Value of Baseline Systemic Immune-Inflammation Index in Advanced Intrahepatic Cholangiocarcinoma Treated with First-Line Gemcitabine–Cisplatin Plus PD-L1 Inhibitor: A Single-Center Retrospective Study
Source: Curr Oncol. 2026 Feb 19;33(2):123. doi: 10.3390/curroncol33020123 (PMC12939202; doi:10.3390/curroncol33020123)

Supplementary Figure S1. Forest plot of multivariable logistic regression for factors associated with objective response (CR/PR vs SD/PD)

Variables	OR (95%CI)	P
Intercept	3.92 (1.40 ~ 11.01)	0.009
Sex		
female	1.00 (Reference)	
male	0.77 (0.32 ~ 1.88)	0.569
CA199 16		
<400	1.00 (Reference)	
⩾400	1.61 (0.62 ~ 4.22)	0.331
Maximumtumordiameter 20		
＜7CM	1.00 (Reference)	
⩾7CM	1.39 (0.51 ~ 3.79)	0.521
Tumornumber		
Multiple	1.00 (Reference)	
Solitary	2.39 (0.94 ~ 6.07)	0.066
Distantmetastasis		
NO	1.00 (Reference)	
YES	1.77 (0.71 ~ 4.43)	0.221
Differentiation		
Medium-to-high	1.00 (Reference)	
Poor	0.82 (0.34 ~ 1.94)	0.647
SIIGROUP		
HIGH	1.00 (Reference)	
LOW	0.02 (0.01 ~ 0.06)	<.001

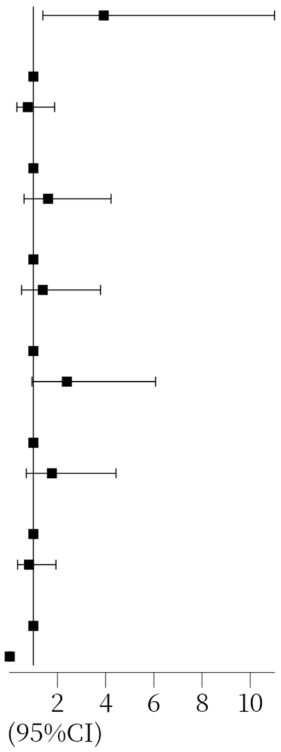

Supplement: Supplementary file 1 [file curroncol-33-00123-s001.zip › curroncol-4054295-supplementary.pdf]
